# Historical faunal exchange between the Pontocaspian Basin and North America

**DOI:** 10.1002/ece3.5602

**Published:** 2019-08-30

**Authors:** Justine Vandendorpe, Christiaan G. C. van Baak, Björn Stelbrink, Diana Delicado, Christian Albrecht, Thomas Wilke

**Affiliations:** ^1^ Department of Animal Ecology and Systematics Justus Liebig University Giessen Giessen Germany; ^2^ CASP Cambridge UK

**Keywords:** dispersal, *Ecrobia*, Hydrobiidae, molecular clock, Pleistocene, Pliocene

## Abstract

*Ecrobia* is a genus of small brackish‐water mud snails with an amphi‐Atlantic distribution. Interestingly, the species occurring in the northwestern Atlantic, *Ecrobia truncata*, is more closely related to the Pontocaspian taxa, *Ecrobia grimmi* and *Ecrobia maritima*, than to the species occurring in the northeastern Atlantic and Mediterranean Sea. At least three colonization scenarios may account for this peculiar biogeographical pattern: (1) a recent human‐mediated dispersal, (2) a historical transatlantic interchange, and (3) a historical transpolar interchange. To test these three scenarios, we used five operational criteria—time of species divergence, first appearance in the fossil record, dispersal limitation as well as environmental filtering and biotic interactions along the potential migration routes. Specifically, we inferred a time‐calibrated molecular phylogeny for *Ecrobia* and reconstructed a paleogeographical map of the Arctic Ocean at 2.5 million years ago (Mya). Based on the five operational criteria, scenarios 1 and 2 can likely be rejected. In contrast, all criteria support scenario 3 (historical transpolar interchange). It is therefore suggested that a bird‐mediated and/or ocean current‐mediated faunal interchange via the Arctic Ocean occurred during the Late Pliocene or Early Pleistocene. This dispersal was likely facilitated by reduced distances between the Eurasian and North American/Greenland landmasses, marine introgressions, and/or a stepping‐stone system of brackish‐water habitats in northern Siberia, as well as a lack of competition along the migration route. As for the direction of dispersal, the scientific data presented are not conclusive. However, there is clearly more support for the scenario of dispersal from the Pontocaspian Basin to North America than vice versa. This is the first study providing evidence for a natural faunal exchange between the Pontocaspian Basin and North America via the Arctic Ocean.

## Graphical abstract

Species of the brackish‐water mud snail genus *Ecrobia* show a peculiar Pontocaspian‐North American sister‐group relationship. At least three colonization scenarios may account for this biogeographical pattern. Based on five operational criteria, two of these scenarios can be rejected and a bird‐ and/or ocean current‐mediated faunal interchange via the Arctic Ocean during the Late Pliocene or Early Pleistocene has to be assumed (“historic transpolar interchange”). This is the first study providing evidence for a natural faunal exchange between the Pontocaspian Basin and North America via the Arctic Ocean.

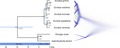

## 1 INTRODUCTION

The brackish‐water mud snail genus *Ecrobia* (=*Ventrosia*) has been extensively used as a model system to study important evolutionary and ecological concepts such as nonadaptive radiation (Davis, 1993), freezing tolerance (Hylleberg & Siegismund, 1987), character displacement (Fenchel, 1975), bird‐mediated long‐distance dispersal (Haase, Naser, & Wilke, 2010), parasite‐induced behavioral changes (Kamiya & Poulin, 2012), and castrate parasitism (Kube, Kube, & Bick, 2006).

Despite this model character of *Ecrobia*, the phylogeography of the taxon remains surprisingly little understood. For instance, it is known that *Ecrobia* has an amphi‐Atlantic distribution, yet exact species ranges are poorly defined. In the western part of its range, *Ecrobia* likely occurs from the Hudson Bay in Canada (Layton, Martel, & Hebert, 2014) to the Chesapeake Bay in the United States (Davis, McKee, & Lopez, 1989). The eastern distribution border ranges from brackish water bodies in the Lake Issyk‐Kul area (Wilke & Delicado, 2019) via the Caspian Sea (Starobogatov, 1970; Wesselingh et al., 2019) to southern Iraq (Haase et al., 2010) and potentially to areas just north of the Arabian (Persian) Gulf (Glöer & Pešić, 2012). The highest species richness of *Ecrobia* can be found in the Mediterranean Basin (Wilke & Delicado, 2019).

Based on these distribution patterns, it would be expected that the Pontocaspian taxa, *Ecrobia grimmi* and *Ecrobia maritima*, are closely related to the Mediterranean *Ecrobia ventrosa* and *Ecrobia spalatiana*. However, Haase et al. (2010) suggested that the North American *Ecrobia truncata* is the sister species of the Pontocaspian taxa. This peculiar close relationship of Pontocaspian and North American species may be explained by at least three biogeographical scenarios: (1) a recent human‐mediated dispersal (human‐mediated transport), (2) a historical dispersal via the Mediterranean Sea/northern Atlantic (transatlantic interchange), and (3) a historical dispersal across the Arctic Ocean (transpolar interchange).

As for the first scenario, there are numerous records of human‐mediated dispersals of Pontocaspian taxa to North America and vice versa, starting as early as the thirteenth century (Petersen, Rasmussen, Heinemeier, & Rud, 1992). Since 1985, 70% of the species that have invaded North America are native to the Pontocaspian Basin (Ricciardi & MacIsaac, 2000). Thus, *Ecrobia* specimens could have been transported in either direction, for example, as larvae in ballast waters of ocean vessels.

The second scenario considers historical dispersal across the Mediterranean Sea/northern Atlantic by either current‐mediated transport of veliger larvae (Scheltema, 1966, 1971) and rafting individuals (Thiel & Haye, 2006), or bird‐mediated transport (stepping‐stone or long‐distance dispersal). In fact, mud snails may be carried in the gut of birds (Morkūnė, Lesutienė, Morkūnas, & Barisevičiūtė, 2018; van Leeuwen, Velde, Lith, & Klaassen, 2012; Wada, Kawakami, & Chiba, 2012) and fish (Aarnio & Bonsdorff, 1997) and are able to survive this passage through the digestive system. In addition, van Leeuwen and van der Velde (2012) suggested passive dispersal by water birds, in which young individuals or eggs are attached to feathers, feet, or bill.

The third scenario assumes that *Ecrobia* individuals have crossed the Arctic Ocean via bird‐mediated or ocean current‐mediated transport, potentially aided by sea level low stands and/or marine introgressions (sensu Barnes, 1988; Dooh, Adamowicz, & Hebert, 2006). In fact, several connections between the Pontocaspian Basin and the Arctic Ocean have been proposed across geological time frames, such as during the Late Miocene (McLaren, 1960), Pliocene (Arnason et al., 2006; Palo & Väinölä, 2006; Richards et al., 2018), and Early Pleistocene (Dooh et al., 2006). Moreover, several authors suggested that the Caspian Sea was colonized by Arctic estuarine taxa during major Pleistocene glaciations or immediately thereafter (Audzijonyte, Damgaard, Varvio, Vainio, & Väinölä, 2005; Davies, 1958; Filippov et al., 2000; Lowry & Stoddart, 1993; Väinölä, Vainio, & Palo, 2001; Zenkevitch, 1963).

Our working hypothesis follows scenario 3 and assumed a transpolar exchange of *Ecrobia* individuals, even though dispersal of brackish water taxa between North America and central Asia across the Arctic has not been reported before. To test this hypothesis in the context of the three scenarios proposed, we combined genetic and fossil data for the mud snail genus *Ecrobia* with ecological, paleogeographical, and biogeographical information. As test statistics, we used a set of five operational criteria (for details see Discussion), involving evolutionary (i.e., time frame of species divergence, age of fossil records) and community characteristics (i.e., dispersal limitations, environmental filtering, biotic interactions). We also composed a paleogeographical map of the Arctic Ocean at 2.5 million years ago (Mya) to infer potential changes in coastlines that may have aided dispersal between populations occurring in North America and the Pontocaspian Basin.

Our study might be of relevance for marine biologists and systematists interested in this ecologically and evolutionary important taxon, for biogeographers studying long‐range dispersal processes of aquatic organisms in space and time, and for (paleo)geographers investigating the biological consequences of past environmental changes.

## 2 MATERIALS AND METHODS

### 2.1 Materials

**Figure 1:**
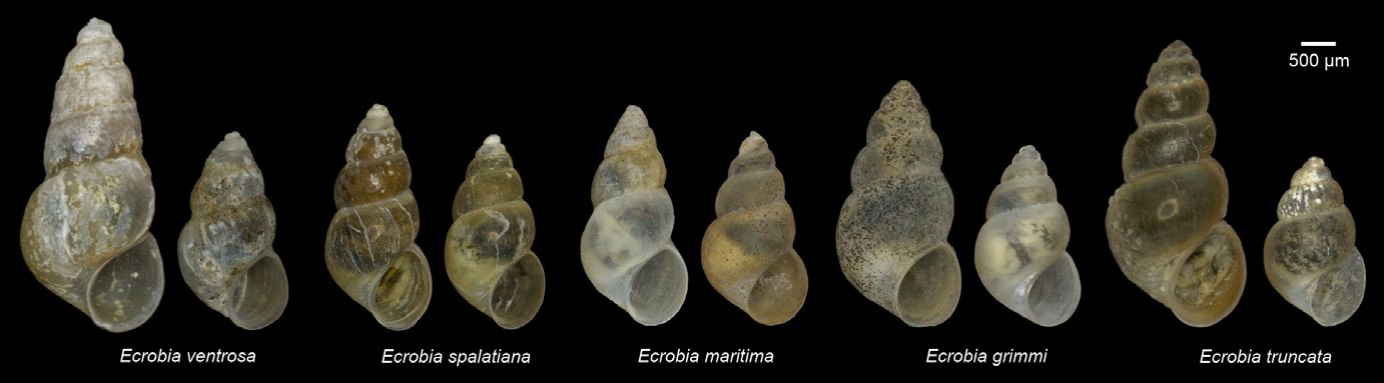
Photographs of ethanol‐preserved specimens of *Ecrobia* spp.

This study includes 67 individuals, representing the distribution ranges of all accepted species of *Ecrobia* (Figure 1). They were collected between 1985 and 2017 from 55 localities (Table A1 in Appendix 1) as part of this or related studies. All specimens were hand‐picked and preserved in 80% ethanol in the field. Surveys were conducted in concordance with CBD regulations (“Nagoya Protocol”). Voucher material and DNA samples were deposited in the University of Giessen Systematics and Biodiversity Collection (Diehl, Jauker, Albrecht, Wilke, & Wolters, 2018) in Germany.

### 2.2 DNA isolation, amplification, and sequencing

DNA was isolated using the CTAB protocol described in Wilke, Davis, Qiu, and Spear (2006). Two mitochondrial markers, the cytochrome *c* oxidase subunit I (COI) and the large subunit rRNA (16S) genes, were amplified using the primers LCO1490 and HCO2198 (Folmer, Black, Hoeh, Lutz, & Vrijenhoek, 1994) and 16Sar‐L and 16Sbr‐H (Palumbi et al., 1991), respectively. PCR amplification was performed with an initial denaturation step at 95°C for 1 min, followed by 35 amplification cycles (denaturation at 95°C for 30 s, annealing at 52°C for 30 s, and elongation at 72°C for 30 s), and a final elongation step at 72°C for 3 min. Bidirectional Sanger sequencing was either conducted on a Long Read IR2 4200 (LI‐COR) sequencer or an ABI 3730 XL (Life Technologies).

The 16S rRNA sequences were aligned with AliView 1.23 (Larsson, 2014), using the secondary structure model for the family Hydrobiidae suggested by Wilke et al. (2013). The protein‐coding COI sequences, which do not contain insertions and deletions in the Hydrobiidae, were unambiguously aligned using the software package BioEdit 7.2.5 (Hall, 1999). As the first base pairs behind the 5′ end of each primer were difficult to read, the fragments were trimmed, resulting in 638‐ and 505‐bp long overlapping fragments for the COI and 16S genes, respectively.

### 2.3 Molecular‐clock analyses

Prior to the molecular‐clock analyses, best‐fit substitution models for the COI and 16S data sets were selected using jModelTest 2 (Darriba, Taboada, Doallo, & Posada, 2012; Guindon & Gascuel, 2003) based on the Akaike information criterion (AIC). The models suggested for the COI and 16S partitions were HKY+Γ and GTR+I+Γ, respectively. Molecular‐clock analyses were then performed using *BEAST (Zhang, Ogilvie, Drummond, & Stadler, 2018) as implemented in BEAST 1.8.4 (Drummond, Suchard, Xie, & Rambaut, 2012). As outgroups, we used two other members of the subfamily Hydrobiinae, that is, *Peringia ulvae* and *Salenthydrobia ferrerii* (Table A1 in Appendix 1). Note that we did not test for substitutional saturation as both genes are not considered to be saturated within the Hydrobiidae (Wilke et al., 2001, 2013). For calibrating the molecular clock, we used two independent means. First, the beginning and end of the Messinian salinity crisis (MSC), which occurred ca. 5.96–5.33 Mya (see Krijgsman, Hilgen, Raffi, Sierro, & Wilson, 1999), were used as lower and upper bounds to time‐calibrate the split between *P. ulvae* and *S. ferrerii* (see Wilke, Schultheiß, & Albrecht, 2009). In addition, the substitution rate of the COI partition was constrained using the marker‐ and trait‐specific Protostomia clock rate of 1.24%–1.57% My^−1^ (percentage of substitutions per lineage per million years) for the HKY+Γ model as suggested by Wilke et al. (2009).

Two independent replicates for both strict‐ and relaxed‐clock analyses were run for 40,000,000 generations each, sampling every 2,000th generation and using the birth–death model (Gernhard, 2008) as species tree prior. Convergence of parameters was ensured in Tracer 1.5 (Rambaut & Drummond, 2007), revealing ESS values for all parameters being >200. Replicates were combined using LogCombiner (BEAST package, 50% burn‐in). The maximum clade credibility (MCC) tree was identified using TreeAnnotator (BEAST package, no additional burn‐in). Strict‐ and relaxed‐clock analyses were compared using the Bayes factor (BF) analysis as implemented in Tracer 1.5 by running 1,000 bootstrap replicates. The BF analysis slightly favored the relaxed‐clock analysis over the strict‐clock analysis (ln P relaxed: −3,794.0, ln P strict: −3,798.9, BF = 2.10). The posterior distributions of the strict‐ and relaxed‐clock analyses were visualized in DensiTree 2.2.5 (Bouckaert, 2010), which is part of the BEAST 2.4.3 package (Bouckaert et al., 2014).

### 2.4 Paleogeographical reconstruction

In order to assess potential historical dispersal events of brackish‐water taxa, it is important to have a basic understanding of the paleogeography and paleocoastline locations. As preliminary analyses indicated that the split between the North American and Pontocaspian *Ecrobia* taxa may have occurred during the Late Pliocene or Early Pleistocene (Wilke, 2003), we created a paleogeographical map of the coastlines for this time period based on various sources. These included Knies et al. (2014) for the width between Greenland and Svalbard, Butt, Drange, Elverhøi, Ottera, and Solheim (2002) for the emerged Barents Sea region, Torsvik, Carlos, Mosar, Cocks, and Malme (2002) for the Late Pliocene North Atlantic coastline, and Vinogradov (1967), Richards et al. (2018), and van Baak et al. (2019) for the Black Sea and Caspian Sea paleogeography. A reconstruction of the tectonically active Mediterranean Sea was not attempted.

## 3 RESULTS

### 3.1 Phylogenetic inference and molecular‐clock analyses

**Figure A1:**
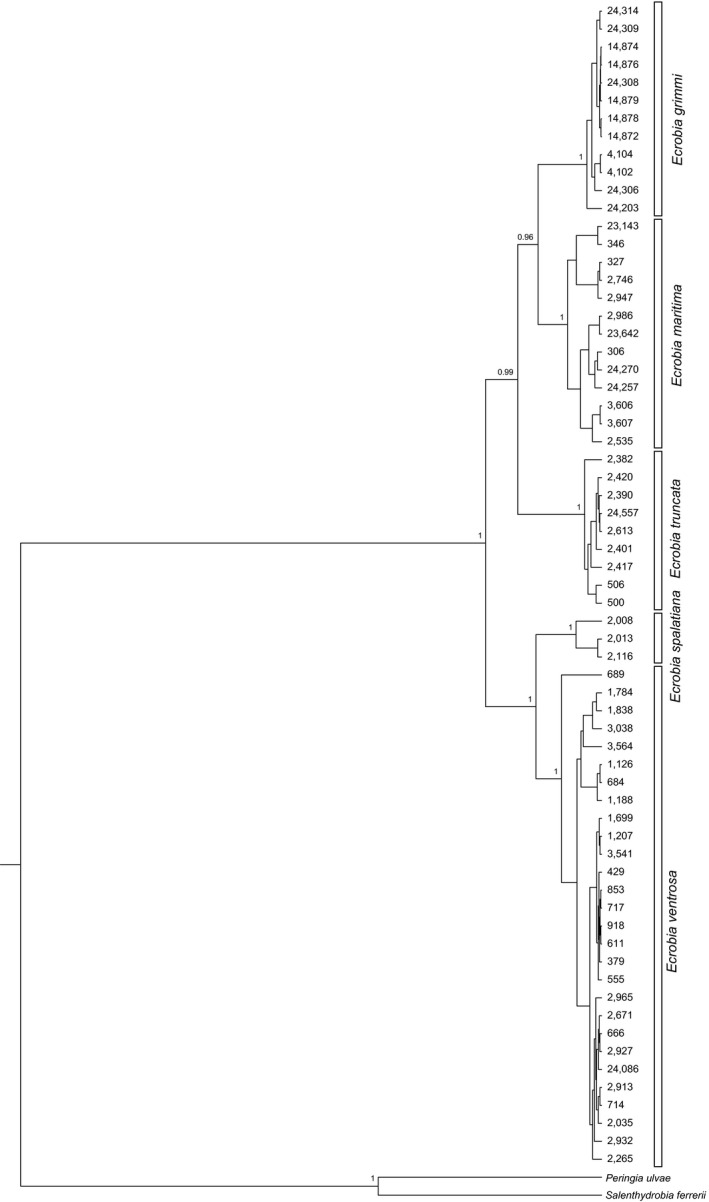
Relaxed‐clock *BEAST tree based on 67 specimens of *Ecrobia* spp. and two outgroup species. BPP values are only provided for deeper nodes (i.e., species level and above)

Tree likelihoods, Bayesian posterior probabilities (BPP), and estimated divergence times were very similar between the species trees inferred by *BEAST under the strict‐ and the relaxed‐clock models. The underlying gene tree for the favored relaxed‐clock model based on all 67 *Ecrobia* specimens is provided as Figure A1 in Appendix 2.

**Figure 2:**
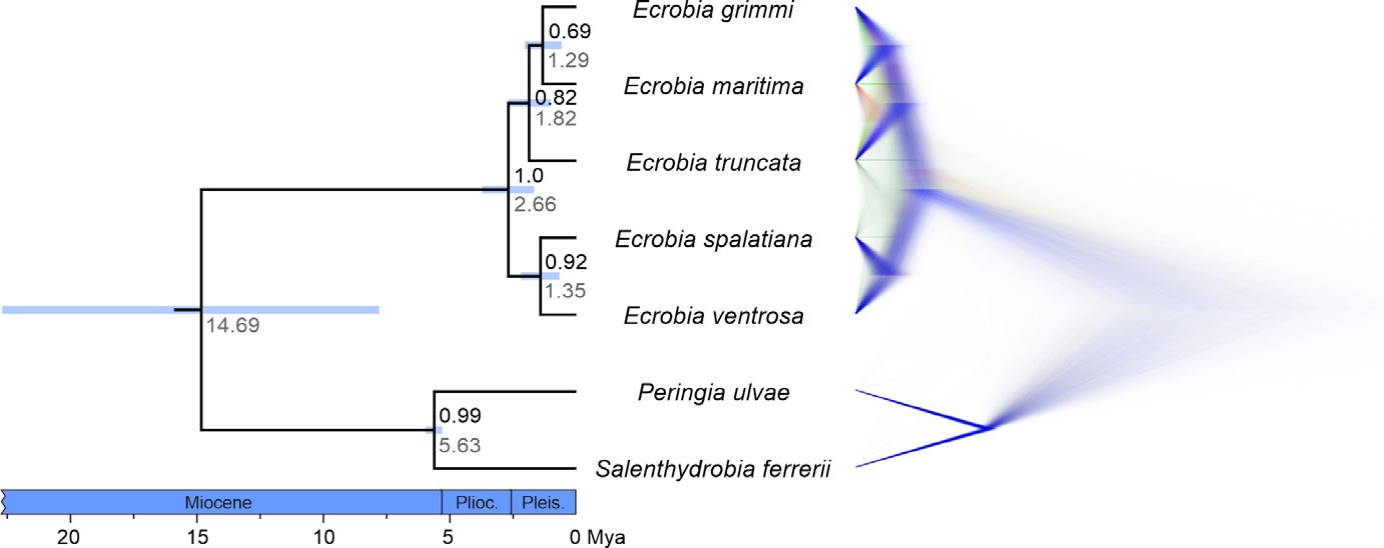
Species trees based on relaxed‐clock *BEAST analyses for *Ecrobia* spp. and two outgroup species. Left: Maximum clade credibility tree with posterior probabilities (black numbers), mean ages in Mya (gray numbers) and respective 95% HPD (blue bars). Right: DensiTree visualization of the *BEAST posterior distribution. The most popular tree is blue, the next most popular red, the third most popular green, and the rest is dark green

The molecular‐clock analyses indicated an age for the split between the North American and Pontocaspian taxa under the favored relaxed‐clock model of ca. 1.82 Mya (95% highest posterior density, HPD: 0.96–2.69 Mya, Figure 2). Similarly, the strict‐clock analysis suggested that this split has occurred ca. 1.87 Mya (1.01–2.69 Mya). Ages for the split between the Mediterranean species (*E. ventrosa* and *E. spalatiana*) and the three other taxa (*E. truncata*, *E. grimmi*, and *E. maritima*) were ca. 2.66 Mya (1.56–3.73 Mya) and ca. 2.69 Mya (1.68–3.73 Mya) under the relaxed‐ and strict‐clock models, respectively.

The relaxed‐clock species tree (Figure 2 left) showed that the North American *E. truncata* is sister to the Pontocaspian taxa *E. grimmi* and *E. maritima*. However, the support is relatively low (BPP 0.82) and the DensiTree (Figure 2 right) indicates that *E. truncata* may alternatively cluster with *E. grimmi*. Sister to the North American/Pontocaspian clade is the relatively well‐supported (BPP 0.92) group including *E. ventrosa* and *E. spalatiana*, which occur in the Mediterranean Basin and bordering seas and oceans.

### 3.2 Paleogeographical reconstruction

**Figure 3:**
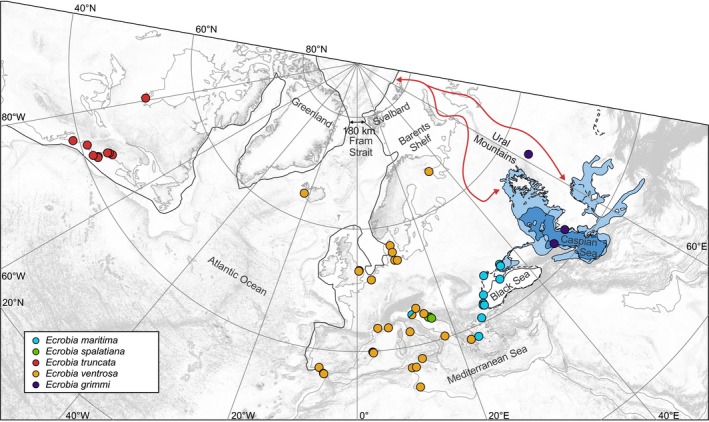
Paleogeographic reconstruction of North Atlantic and Pontocaspian coastlines during the Early Pleistocene, ca. 2.5 Mya (dark gray lines), superimposed on the present‐day geography (light gray lines). The red arrows indicate potential pathways along which a Late Pliocene–Early Pleistocene marine connection between the Arctic Ocean and the Caspian Sea may have existed. Dots represent sampled localities for *Ecrobia* spp. according to Table A1 in Appendix 1. Projection: Polar stereographic

Northern Hemisphere glaciations played an important role in shaping the landscape at high northern latitudes during the last ~1 My. Erosion at the base of these ice sheets was significant and essentially stripped the continent from its sedimentary cover. As such, preexisting higher elevations were significantly lowered (Figure 3). This is most notable in the Barents Sea shelf, which at present is submerged and has an average depth of 230 m. Redistributing the sediment volume at the bottom of the ocean in front of what would have been the Barents ice sheet back onto this shallow marine shelf indicates that prior to 1 Mya the Barents Shelf must still have been largely continental (Butt et al., 2002). This means that the coastline of the North Atlantic would have formed an almost straight line from present‐day northern Norway to Svalbard.

At present, the narrowest point between Svalbard and Greenland is less than 450 km. This passage is known as the Fram Strait. The middle part of the Fram Strait is the northern part of the actively spreading Mid‐Atlantic Ridge. As such, the Fram Strait would have been even narrower in the geological past. Estimates for 2–3 Mya indicate the width would have been around 180 km (Torsvik et al., 2002, also see Figure 3).

In addition to the North Atlantic being narrower, differences in the Caspian Sea region are evident, which would have benefitted dispersal of *Ecrobia* spp. between the Caspian Sea and the Arctic Ocean. Because of the low topography to the north of the Caspian Sea, increases in water level cause vast expansions of the sea surface area. During the Late Pliocene/Early Pleistocene, such a major expansion pushed the northern coastline of the Caspian Sea about 1,000 km further north than at present (Richards et al., 2018; van Baak et al., 2019). At the same time, there might have been a marine transgression from the Arctic Ocean toward the Caspian Sea on either side of the Ural Mountains (Krijgsman et al., 2019, also see Figure 3). However, due to glacial erosion in the last ~1 My (see above), the geological record in this crucial region has largely been removed. For this reason, it is extremely difficult to properly reconstruct the paleogeography of the region between the Caspian Sea and the Arctic Ocean.

## 4 DISCUSSION

The main goal of this study was to test for three biogeographical scenarios that could potentially account for the peculiar sister‐group relationship of Pontocaspian and North American mud snails (*Ecrobia* spp.). These scenarios are (1) a recent human‐mediated dispersal (human‐mediated transport), (2) a historical dispersal via the Mediterranean Sea/northern Atlantic (historical transatlantic interchange), and (3) a historical dispersal across the Arctic Ocean (historical transpolar interchange). As operational criteria, we used time of species divergence, first appearance in the fossil record, dispersal limitation as well as environmental filtering and biotic interactions along the potential migration route. Our working hypothesis assumed a transpolar exchange of *Ecrobia* individuals.

### 4.1 Support for biogeographical scenarios

Based on our operational criteria (Table 1), scenario 1 (human‐mediated transport) can be dismissed. First, the split between the North American and Pontocaspian species (ca. 1.0–2.7 Mya, Figure 2) clearly predates the earliest human migrations ca. 25,000 years ago (Price, 2018). Second, *Ecrobia* spp. are known from the North American and Pontocaspian fossil records since the Pleistocene (Spencer & Campbell, 1987) and Middle Miocene (Büyükmeriç, Wesselingh, & Alçiçek, 2016), respectively. Therefore, the North America‐Pontocaspian faunal interchange of *Ecrobia* specimens was very likely mediated by natural (non‐human) mechanisms.

**Table 1:** Operational criteria for three colonization scenarios accounting for the sister‐group relationship of Pontocaspian and North American *Ecrobia* spp.

Scenario	Operational criterion				
	Time of species divergence	Fossil record	Dispersal limitation (dispersal distance)	Environmental filtering (suitable habitats along migration route)	Biotic interactions (competition along migration route)
1) Human‐mediated transport	−	−	N/A	N/A	N/A
2) Historical transatlantic interchange	+	+	+/−	+	−
3) Historical transpolar interchange	+	+	+	+	+

Note

−, scenario not supported; +, scenario supported; N/A, not applicable.

Scenario 2 (historical transatlantic interchange) is supported by both the time frame of divergence between North American and Pontocaspian taxa as well as by the fossil records (see above). Moreover, brackish‐water habitats along the Atlantic coastlines could have served as stepping‐stones for the active or passive dispersal of *Ecrobia* specimens, thus mitigating the effects of environmental filtering. However, the minimum straight‐line ocean distance between the Pontocaspian area and North America during the early Pleistocene via the Atlantic Ocean (see Figure 3) was >7,500 km, thus constituting a considerable dispersal limitation. Moreover, while crossing the Mediterranean Sea/Atlantic Ocean, Pontocaspian species would have encountered considerable competition from congeners (criterion biotic interactions in Table 1). The latter criterion is of particular concern, as the genus *Ecrobia* constitutes a nonadaptive radiation. Within such radiations, biotic interactions will very likely result in competitive exclusion (Gittenberger, 1991; Wilke, Benke, Brändle, Albrecht, & Bichain, 2010). Due to these dispersal limitations and potential biotic interactions, a historical transatlantic interchange seems unlikely.

Scenario 3 (historical transpolar interchange) is supported by both evolutionary criteria—time of species divergence and the earliest available fossil records (see above, Table 1). It is also supported by the three‐community criteria—dispersal limitations, environmental filtering, and biotic interactions.

Dispersal limitations in the latter scenario were likely considerably lower than in scenario 2. During the Early Pleistocene, the North American/Greenland landmass and the Eurasian landmass were only separated by the narrow Fram Strait (Knies et al., 2014, also see Figure 3). Moreover, between ca. 2.7 and 2.4 Mya, an episode of distinctly more marine conditions occurred in the Pontocaspian Basin (Richards et al., 2018), coinciding with a northward extension of the Caspian Sea (van Baak et al., 2019) and a possible marine transgression from the Arctic Ocean toward the Caspian Sea, driven by isostatic changes due to loading by northern hemisphere ice sheets (Krijgsman et al., 2019; Richards et al., 2018, also see Figure 3).

Alternatively, brackish water lakes on either side of the Ural Mountains may have served as stepping‐stone systems for bird‐mediated dispersal of *Ecrobia* specimens (see Haase et al., 2010, also see the *Ecrobia* location east of the Ural Mountains in Figure 3). Such water bird flyways between the Caspian Sea and the Arctic Ocean still exist today (Boere & Stroud, 2006). Further dispersal along the coastlines of the Arctic Ocean could have been facilitated by bird‐mediated transport (sensu Haase et al., 2010) and larval drift or rafting of adult individuals along the Beaufort Gyre, which has been persisting since the Middle Miocene (Matthiessen, Knies, Vogt, & Stein, 2009). In fact, Thiel and Haye (2006) suggested that *Ecrobia* specimens are able to disperse over several thousand kilometers by rafting. Overall, the dispersal distance in scenario 3 is at least 3,000 km shorter than in scenario 2.

Moreover, habitat suitability along the potential migration route from the Pontocaspian area to the Arctic Ocean (or vice versa) was likely excellent for *Ecrobia* spp. Today, the Pontocaspian *E. grimmi* is widespread across central Asia, occurring in brackish water bodies from the central Ural Mountains in the north to the Persian Gulf in the south and the Lake Issyk‐Kul area in the east (reviewed in Wilke & Delicado, 2019). Thus, environmental filtering appears to have been low. Moreover, environmental temperatures during the Late Pliocene/Early Pleistocene were higher than today and the sea‐ice cover was periodically reduced (Matthiessen et al., 2009; Melles et al., 2012). In addition, the Barents Sea shelf was still exposed (e.g., Butt et al., 2002), providing ample space for brackish‐water habitats. This may have further mitigated potential effects of environmental filtering.

Finally, and most importantly, there is no evidence for the occurrence of other species of *Ecrobia* along the potential migration route for a transpolar interchange. As biotic interactions (i.e., species competition) play a major role in the distribution of members of nonadaptive radiations (Wilke et al., 2010), the absence of this strong filter likely facilitated the spread of *Ecrobia* sp. across the Arctic Ocean.

Combining all pieces of evidence derived from our operational criteria, the most parsimonious explanation for the faunal interchange of North American and Pontocaspian mud snails would be a historical transpolar dispersal. Thus, our working hypothesis cannot be rejected. To our best knowledge, this is the first evidence for such an interchange dating back to the Late Pliocene/Early Pleistocene.

### 4.2 Direction of faunal interchange

Whereas our study provides strong evidence for a historical interchange of North American and Pontocaspian mud snails, the direction of dispersal, that is, North America to the Pontocaspian Basin or vice versa, remains to be answered.

Considering natural dispersal events between the Eurasian part of the Arctic Ocean and the Pontocaspian Basin between 1.8 and 0.01 Mya, there are at least 24 Arctic invertebrate taxa that have entered the Pontocaspian Basin from the north with glacial meltwaters (Orlova, 2000). The well‐studied Caspian seal, for example, is most likely the descendant of ancestors inhabiting the polar seas (Arnason et al., 2006; Davies, 1958; Fulton & Strobeck, 2010; McLaren, 1960; Palo & Väinölä, 2006). These findings would lend some support to the assumption that the Pontocaspian Basin was the sink and not the source for the dispersal of *Ecrobia* mud snails.

However, considering the dispersal route and the entire body of evidence, the picture changes. (1) Acknowledging an incomplete *Ecrobia* fossil record and problems with fossil identifications, the record of *Ecrobia* in the Pontocaspian Basin is considerably older than the one in North America (see above), indicating dispersal from the Pontocaspian Basin to North America. (2) The current center of *Ecrobia* biodiversity is the western Mediterranean/Pontocaspian region, supporting the assumption that this area has also been the ancestral area for the spread of *Ecrobia* spp. (3) The prevailing, wind‐driven direction of the Beaufort Gyre in the Arctic Ocean is clockwise, facilitating larval drift or rafting of adult individuals from the Siberian coasts of the Arctic Ocean to the North American coasts. In addition, the Transpolar Drift Stream in the Arctic Ocean transports runoffs from Siberian rivers either to Greenland or Canada (Emery, Fowler, & Maslanik, 1997). (4) In the DensiTree (see Figure 2 right), the Pontocaspian species *E. grimmi* and *E. maritima* cluster apart from the North American *E. truncata*. Such a pattern would be in line with both the Pontocaspian being a sink and source. However, alternatively (and less likely) the Pontocaspian *E. grimmi* clusters with the North American *E. truncata* and not with the other Pontocaspian species—*E. maritima* (see the red branches in Figure 2 right). Such an alternative topology would be difficult to explain with North America being the sink for Pontocaspian *Ecrobia* spp. (5) Finally, in order to disperse from North America to Eurasia, the common ancestor of *E. truncata*, *E. grimmi*, and *E. maritima* would have had to enter North America from Europe in the first place, requiring another trans‐ocean dispersal step, rendering this scenario less parsimonious.

Combining the pieces of evidence provided above, we think that dispersal of mud snails from the Pontocaspian Basin (or brackish‐water systems in northern Siberia) to North America provides a straightforward and parsimonious explanation for the peculiar biogeographical patterns seen in *Ecrobia* spp.

### 4.3 Limitations of our study

A potential limitation of our study is the lack of nuclear markers. We did amplify the internal transcribed spacer 2 (ITS2) for 46 individuals of *Ecrobia* spp. However, the resulting phylogenetic tree was unresolved due to incomplete lineage sorting. Moreover, when combining this nuclear dataset with our mitochondrial dataset, support values in the phylogenetic trees did not improve. However, the major conclusions of this paper are drawn from a combination of molecular, fossil, and biogeographical information, with the individual findings being broadly in accordance. Therefore, we believe that additional nuclear data would not have changed the overall outcome of this study.

Moreover, due to the unfavorable ratio of only five studied species but four distribution areas (i.e., Mediterranean Sea, Eastern Atlantic, Western Atlantic, and Pontocaspian), a meaningful ancestral area reconstruction for *Ecrobia* spp. is not possible, which could potentially have helped to unravel the direction of dispersal. However, the indirect evidence provided above partly compensates for the lack of such an analysis. In addition, an ancestral area reconstruction would not have had a direct effect on the outcome of our scenario testing, which favors scenario 3—a historical transpolar interchange of mud snails.

## 5 CONCLUDING REMARKS

The purpose of our study was to test for three colonization scenarios that could potentially account for the peculiar distribution pattern of *Ecrobia* spp., that is, a sister‐group relationship of North American and Pontocaspian taxa, using five operational criteria: time of species divergence, first appearance in the fossil record, dispersal limitation as well as environmental filtering and biotic interactions along the potential migration route. Scenario 1 (human‐mediated transport) can be dismissed based on time of divergence and age of fossil records. Scenario 2 (historical transatlantic interchange) also appears to be unlikely due to considerable dispersal limitations and biotic interactions along a potential dispersal route. In contrast, all five operational criteria support scenario 3 (historical transpolar interchange). We suggest that the faunal interchange occurred during the Late Pliocene or Early Pleistocene. Ocean current‐ or bird‐mediated dispersal was likely facilitated by reduced distances between the North American/Greenland and Eurasian landmasses, marine introgressions, and/or a stepping‐stone system of brackish‐water habitats in northern Siberia as well as a lack of competition along the migration route. The data presented are not conclusive for the direction of dispersal, that is, from North America to the Pontocaspian Basin or vice versa. However, there is clearly more evidence supporting the latter scenario. We therefore conclude that a dispersal from the Pontocaspian Basin to North America gives a more parsimonious explanation for the patterns observed.

This is the first time that a faunal connection between the Pontocaspian Basin and North America is explained by a natural transpolar dispersal event. It thus sheds new light on the biogeography of brackish‐water taxa in the Northern Hemisphere and on the consequences of past environmental and geological changes.

## CONFLICT OF INTEREST

None declared.

## AUTHOR CONTRIBUTIONS

JV and TW designed the study with contributions of CGCvB, BS, DD and CA. JV, CGCvB and BS conducted the analyses. JV and TW wrote the manuscript with contributions of CGCvB, BS, DD and CA.

## Data Availability

All data used in the analyses are available from GenBank (for GenBank accession numbers see Table A1 in Appendix 1).

## APPENDIX 1

**Table A1:** Locality information, DNA voucher numbers, and GenBank accession numbers for specimens of *Ecrobia* spp. and two outgroup species

Species	Collection site	DNA voucher no.	GenBank accession no.	
			COI	16S
*Ecrobia grimmi* (Clessin & Dybowski, 1888)	Kazakhstan, near Karakol Lake (43.4677°N, 51.3106°E)	24203	MN167715	MN167766
		24306	MN167716	MN167767
		24308	MN167717	MN167768
		24309	MN167718	MN167769
		24314	MN167719	MN167770
	Russia, Salamatka Lake (55.2111°N, 62.0253°E)	14872	MN167720	MN167771
		14874	MN167721	MN167772
		14876	MN167722	MN167773
		14878	MN167723	MN167774
		14879	MN167724	MN167775
	Russia, Sulak Bay (43.3010°N, 47.5180°E)	4102	GQ505913 a	MN167776
		4104	MN167725	MN167777
*Ecrobia maritima* (Milaschewitsch, 1916)	Bulgaria, bay near Nessebar (42.66°N, 27.72°E)	346	AF253076 b	MN167778
	Bulgaria, bay near Strandscha (42.430°N, 27.513°E)	306	AF449216 c	AF478403 c
	Bulgaria, Rapotamo River Estuary (42.325°N, 27.752°E)	327	MN167726	MN167779
	Greece, Evros Delta (40.769°N, 26.054°E)	2535	MN167727	MN167780
	Greece, Kimi Aliveri (38.3919°N, 24.0159°E)	3606	MN167728	MN167781
		3607	MN167729	MN167782
	Italy, Po Estuary (44.83°N, 12.27°E)	2746	MN167730	MN167783
	Romania, Mangalia (43.8042°N, 28.5917°E)	23143	MN167731	MN167784
	Ukraine, Molochnyi Liman (46.6°N, 35.3°E)	2947	MN167732	MN167785
	Ukraine, Odessa, Hydrological Station (46.4416°N, 30.7726°E)	23642	MN167733	MN167786
	Ukraine, Sevastopol (44.61°N, 33.45°E)	2986	AY616139 d	MN167787
	Ukraine, Utlyukskij Liman (46.3026°N, 35.2772°E)	24257	MN167734	MN167788
	Ukraine, Utlyukskij Liman (46.2682°N, 35.2664°E)	24270	MN167735	MN167789
*Ecrobia spalatiana* (Radoman, 1973)	Croatia, Pontana Spring (43.5293°N, 16.2767°E)	2013	MN167736	MN167790
	Croatia, Pirovac Spring (43.8167°N, 15.6832°E)	2116	MN167737	MN167791
	Croatia, Krka River Estuary (43.8172°N, 15.9282°E)	2008	MN167738	MN167792
*Ecrobia truncata* (Vanatta, 1924)	Canada, Longridge Camp (51.7999°N, 80.6643°W)	24557	MN167739	MN167793
	USA, Menemsha Pond (41.3434°N, 70.7654°W)	2401	MN167740	MN167794
	USA, Damariscotta River (44.20°N, 69.50°W)	2417	MN167741	MN167795
	USA, Duck Creek (41.9368°N, 70.0290°W)	2382	MN167742	MN167796
	USA, Flax Pond (40.963°N, 73.141°W)	506	MN167743	MN167797
	USA, Long Point Marshes (42.035°N, 70.195°W)	2420	MN167744	MN167798
	USA, north of Burnett Road Bridge (43.825°N, 70.076°W)	2390	MN167745	MN167799
	USA, near Wetlands Institute (39.06°N, 74.77°W)	500	AF449217 c	AF478404 c
	USA, Spurwink River (43.576°N, 70.258°W)	2613	MN167746	MN167800
*Ecrobia ventrosa* (Montagu, 1803)	Croatia, Zrce Beach (44.5421°N, 14.9138°E)	2035	MN167747	MN167801
	Croatia, small bay of the sea (44.5421°N, 14.9136°E)	24086	MN167748	MN167802
	Denmark, Ajstrup Bugt in Mariager Fjord (56.68°N, 10.22°E)	853	AF118353 e	MN167803
	Denmark, Fyn Island, Odense Fjord (55.50°N, 10.53°E)	429	AF118359 e	MN167804
	France, Etang de Villepey (43.3982°N, 6.7291°E)	2932	MN167749	MN167805
	France, canal near Etang de Gines (43.4887°N, 4.4432°E)	2965	MN167750	MN167806
	Germany, Lake Neustadt (54.1067°N, 10.8067°E)	918	AF118363 e	MN167807
	Germany, Boiensdorfer Werder (54.0229°N, 11.5345°E)	379	AF118368 e	MN167808
	Greece, spring east of Itea (38.4273°N, 22.4557°E)	3564	MN167751	MN167809
	Iceland, Gálgahraun (64.476°N, 22.167°W)	555	AF118341 e	MN167810
	Italy, Bambinello Spring (40.283°N, 17.838°E)	2265	MN167752	MN167811
	Italy, Laguna di Grado (45.6865°N, 13.4371°E)	2927	MN167753	MN167812
	Italy, Laguna di Orbetello di Ponente (42.27°N, 11.13°E)	666	AF118326 e	MN167813
	Italy, Po DelSacca di Goro (44.83°N, 12.27°E)	2671	MN167754	MN167814
	Italy, Saline Ettore Infersa (37.88°N, 12.45°E)	3038	MN167755	MN167815
	Russia, Lagoon “Levin navolok” (66.32°N, 33.53°E)	611	AF118346 e	MN167816
	Spain, near Punta Umbria (37.255°N, 7.143°W)	1188	MN167756	MN167817
	Spain, San Francisco de Asís Lagoon (36.394°N, 6.137°W)	684	AF118330 e	MN167818
	Spain, mouth of Torrent de la Borges (39.7303°N, 3.2372°E)	3541	MN167757	MN167819
	Spain, Sa Albufereta (39.8617°N, 3.0918°E)	1207	MN167758	MN167820
	Spain, Salina San Miguel (36.46°N, 6.22°W)	1126	MN167759	MN167821
	Spain, within the Port d'Alcúdia (39.836°N, 3.118°E)	1699	MN167760	MN167822
	Tunisia, southern bank of Lac de Tunis (36.8082°N, 10.2629°E)	1838	MN167761	MN167823
	Tunisia, north of Temime (36.7976°N, 11.0319°E)	1784	MN167762	MN167824
	Tunisia, Island Djerba (33.8200°N, 11.0700°E)	689	MN167763	MN167825
	The Netherlands, near Braakman Kreek (51.3358°N, 3.7437°E)	2913	MN167764	MN167826
	United Kingdom, Holme Broadwater (52.977°N, 0.565°E)	714	AF118340 e	MN167827
	United Kingdom, Snettisham RSPB bird reserve (52.86°N, 0.46°E)	717	AF118335 e	AF478402 c
*Peringia ulvae* (Pennant, 1777)	France, Brittany, Finistère, Lostrouc'h (48.634°N, 4.537°E)	1968	MN167765	MN167828
*Salenthydrobia ferrerii* Wilke, 2003	Italy, Bambinello Spring (40.283°N, 17.838°E)	2231	AF449201 c	AF478408 c

a Haase et al. (2010),

b Davis et al. (1998),

c Wilke (2003),

d Kevrekidis, Wilke, and Mogias (2005),

e Wilke and Davis (2000).
